# 1,1′-[(2-Phenyl-2,3-dihydro-1*H*-benz­imidazole-1,3-di­yl)bis­(methyl­ene)]bis­(1*H*-benzotriazole)

**DOI:** 10.1107/S1600536811055486

**Published:** 2012-01-07

**Authors:** Augusto Rivera, Hector Jairo Osorio, Jaime Ríos-Motta, Karla Fejfarová, Michal Dušek

**Affiliations:** a Departamento de Química, Universidad Nacional de Colombia, Ciudad Universitaria, Bogotá, Colombia; b Universidad Nacional de Colombia, Sede Manizales, Colombia; c Institute of Physics ASCR, v.v.i., Na Slovance 2, 182 21 Praha 8, Czech Republic

## Abstract

The imidazole ring in the title compound, C_27_H_22_N_8_, adopts a slight envelope conformation with the C atom carrying the phenyl ring being the flap atom. The phenyl ring is almost perpendicular to the mean plane of the imidazole ring [dihedral angle = 88.90 (7)°]. The (1*H*-benzotriazol-1-yl)methyl groups bound to the imidazole ring are positioned on the same side of the imidazole ring. The dihedral angle between these benzotriazolyl rings is 17.71 (5)°. The crystal packing is stabilized by a C—H⋯π inter­action, which connects the mol­ecules into zigzag chains running along the *b* axis.

## Related literature

For a related structure see: Rivera *et al.* (2011 ▶). For the synthesis of the precursor and the title compound, see: Rivera *et al.* (2000 ▶, 2004 ▶). For ring conformations, see: Cremer & Pople (1975 ▶).
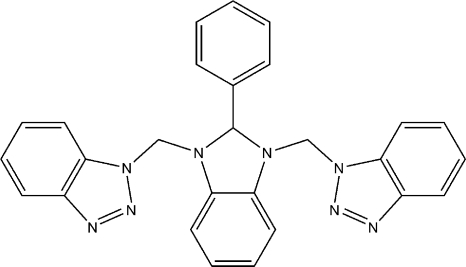


## Experimental

### 

#### Crystal data

C_27_H_22_N_8_
*M* *_r_* = 458.5
Orthorhombic, 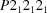

*a* = 9.2721 (2) Å
*b* = 13.6449 (3) Å
*c* = 17.1883 (4) Å
*V* = 2174.61 (8) Å^3^
*Z* = 4
Cu *K*α radiation
μ = 0.70 mm^−1^
*T* = 120 K
0.38 × 0.25 × 0.18 mm

#### Data collection

Agilent Xcalibur diffractometer with an Atlas (Gemini ultra Cu) detector
Absorption correction: multi-scan (*CrysAlis PRO*; Agilent, 2010 ▶) *T*_min_ = 0.325, *T*_max_ = 1
27948 measured reflections
2206 independent reflections
2149 reflections with *I* > 3σ(*I*)
*R*_int_ = 0.030

#### Refinement

*R*[*F* ^2^ > 2σ(*F* ^2^)] = 0.027
*wR*(*F* ^2^) = 0.080
*S* = 1.80
2206 reflections
317 parameters
H-atom parameters constrained
Δρ_max_ = 0.12 e Å^−3^
Δρ_min_ = −0.12 e Å^−3^

### 

Data collection: *CrysAlis PRO* (Agilent, 2010 ▶); cell refinement: *CrysAlis PRO*; data reduction: *CrysAlis PRO*; program(s) used to solve structure: *SIR2002* (Burla *et al.*, 2003 ▶); program(s) used to refine structure: *JANA2006* (Petříček *et al.*, 2006 ▶); molecular graphics: *DIAMOND* (Brandenburg & Putz, 2005 ▶); software used to prepare material for publication: *JANA2006*.

## Supplementary Material

Crystal structure: contains datablock(s) global, I. DOI: 10.1107/S1600536811055486/bt5766sup1.cif

Structure factors: contains datablock(s) I. DOI: 10.1107/S1600536811055486/bt5766Isup2.hkl

Supplementary material file. DOI: 10.1107/S1600536811055486/bt5766Isup3.cml

Additional supplementary materials:  crystallographic information; 3D view; checkCIF report

## Figures and Tables

**Table 1:** Hydrogen-bond geometry (Å, °) *Cg*6 is the centroid of the C15–C20 aromatic ring.

*D*—H⋯*A*	*D*—H	H⋯*A*	*D*⋯*A*	*D*—H⋯*A*
C12—H12⋯*Cg*6^i^	0.96	2.61	3.5597 (19)	169

Symmetry code: (i) 
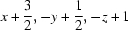
.

**Fig. 1.**
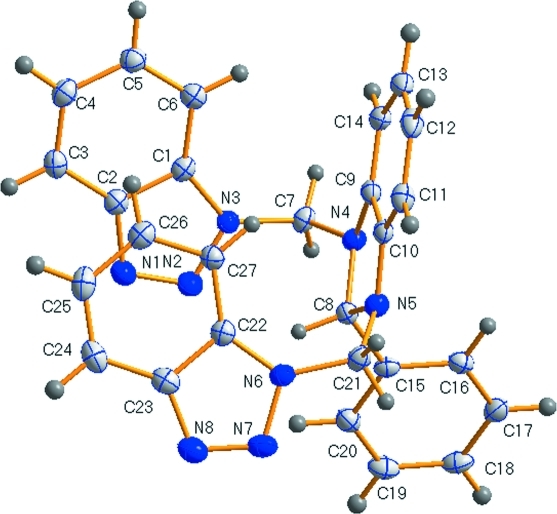
A view of (I) with the numbering scheme. Displacement ellipsoids are drawn at the 50% probability level.

**Fig. 2.**
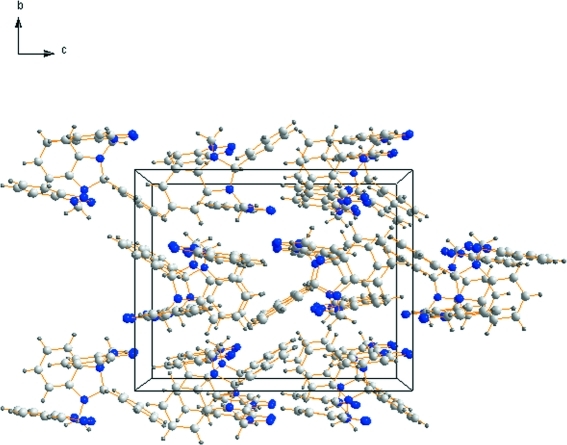
Packing of the molecules of the title compound view along b axis.

## supplementary crystallographic information

### Comment

Considerable work from our laboratory has been concerned with the synthesis of benzotriazol-1-ylmethyl groups attached to imidazolidine-like nitrogen atoms in heterocyclic aminals. The title compound (I) was synthesized *via* route modified from that reported (Rivera *et al.*, 2000) by reaction of 1,1'-(1*H*-benzimidazole-1,3(2*H*)-diyl)bis(methylene)-bis-(1*H*-benzotriazole) with benzaldehyde. The whole procedure is a two-step method with a good overall yield. The starting compound was prepared according to literature procedure (Rivera *et al.*, 2004). The structure of this precursor, whose structure we reported previously (Rivera *et al.*, 2011), showed that the compound exists in a conformation in which the benzotriazol-1-ylmethyl moieties arranged in *anti* disposition with respect to benzimidazolidine ring. In the title compound, the presence of a phenyl substituent on the central carbon of the benzimidazolidine ring may influence the pendant substituent to occupy a *syn* conformation.

Although the molecule potentially exhibits mirror symmetry, in the crystalline state the spatial disposition of two 1*H*-benzotriazol-1-yl)methyl units are not perfectly identical (Figure 1). However, the measured bond lengths and angles are extremely close and consequently only mean values will be cited in this discussion. The interatomic distances and angles of title compound (I) are comparable with a related structure (Rivera *et al.*, 2011). The imidazole ring is an envelope conformation with the central C8 atom being the flap atom as seen in the puckering parameters Q(2) = 0.1259 (16) Å and φ2 = 41.2 (7) ° (Cremer & Pople, 1975). With reference to this plane, the phenyl ring lies to one side of the plane and is almost perpendicular to the mean plane of the heterocyclic ring, with a dihedral angle of 88.898 (66)°. The (1*H*-benzotriazol-1-yl)methyl groups bound to the central heterocyclic ring are almost *syn* as seen in the C7—N4···N5—C21 torsion angle of 9.91 (37)°. This is contrary to what is observed in the related structure (Rivera *et al.*, 2011), where the two (benzotriazol-1-yl)methyl groups are located in an *anti* position with respect to the benzimidazoline moiety. In the title compund the dihedral angle between these benzotriazolyl rings is 17.712 (47)°.

In benzimidazoline ring occurs H12···*Cg*6 = 2.61 (5) Å, which connect the molecules into a chain along the *b* axis (Figure 2), *Cg*6 is the centroid of ring C15—C20.

### Experimental

To a solution in methanol (5 ml) of 1,1'-(1*H*-benzimidazole-1,3(2*H*)-diyl)bis(methylene)-bis-(1*H*-benzotriazole) (0.27 mmol) prepared beforehand following previously described procedures (Rivera *et al.*, 2004), was added benzaldehyde (0.27 mmol) dissolved in methanol (1 ml). The reaction mixture was refluxed with stirring for 1 h. The reaction mixture was allowed to stand for 3 h, at which time a white precipitate was formed, it was filtered, washed and dried. Mp = 453–455 K, yield: 19%.

^1^H NMR (400 MHz, CDCl_3_) δ (p.p.m.): 5.71 (d, *J* = 14.4 Hz, 2H, N—CH_2_—N, benzylic), 5.84 (s, 1H, N—CH—N) 5.84 (d, *J* = 14.4 Hz, 2H, N—CH_2_—N, benzylic), 6.85 (m, 2H, H-2 and H-3), 6.93 (m, 2H, H-4 and H-5), 7.03 (m, 2H, H-8 and H-9), 7.27 (m, 2H, H-15 and H-20), 7.37 (m, 5H, H-10, H-13,H-14, H-15, H-18, H-19 and H-20), 7.57 (td, *J* = 6.8 Hz, *J*^4^ = 1.6 Hz, 2H, H-6 and H-7), 7.93 (d, *J* = 6.4 Hz, 2H, H-12 and H– 17).

### Refinement

All H atoms atoms were positioned geometrically and treated as riding on their parent atoms. The isotropic atomic displacement parameters of hydrogen atoms were set to 1.2×*U*_eq_ of the parent atom. As the structure contains only light atoms, Friedel pairs were merged and the Flack parameter has not been determined.

### Crystal data

**Table:**

C_27_H_22_N_8_	*F*(000) = 960
*M**_r_* = 458.5	*D*_x_ = 1.400 Mg m^−^^3^
Orthorhombic, *P*2_1_2_1_2_1_	Cu *K*α radiation, λ = 1.5418 Å
Hall symbol: P 2ac 2ab	Cell parameters from 20891 reflections
*a* = 9.2721 (2) Å	θ = 3.2–67.0°
*b* = 13.6449 (3) Å	µ = 0.70 mm^−^^1^
*c* = 17.1883 (4) Å	*T* = 120 K
*V* = 2174.61 (8) Å^3^	Prism, colourless
*Z* = 4	0.38 × 0.25 × 0.18 mm

### Data collection

**Table:**

Agilent Xcalibur diffractometer with an Atlas (Gemini ultra Cu) detector	2206 independent reflections
Radiation source: Enhance Ultra (Cu) X-ray Source	2149 reflections with *I* > 3σ(*I*)
mirror	*R*_int_ = 0.030
Detector resolution: 10.3784 pixels mm^-1^	θ_max_ = 67.1°, θ_min_ = 4.1°
Rotation method data acquisition using ω scans	*h* = −11→10
Absorption correction: multi-scan (CrysAlis PRO; Agilent, 2010)	*k* = −16→16
*T*_min_ = 0.325, *T*_max_ = 1	*l* = −19→17
27948 measured reflections	

### Refinement

**Table:**

Refinement on *F*^2^	H-atom parameters constrained
*R*[*F*^2^ > 2σ(*F*^2^)] = 0.027	Weighting scheme based on measured s.u.'s *w* = 1/(σ^2^(*I*) + 0.0016*I*^2^)
*wR*(*F*^2^) = 0.080	(Δ/σ)_max_ = 0.005
*S* = 1.80	Δρ_max_ = 0.12 e Å^−^^3^
2206 reflections	Δρ_min_ = −0.12 e Å^−^^3^
317 parameters	Extinction correction: B-C type 1 Lorentzian isotropic (Becker & Coppens, 1974)
0 restraints	Extinction coefficient: 2500 (500)
88 constraints	

### Special details

**Table:**

Experimental. CrysAlisPro (Agilent, 2010) Empirical absorption correction using spherical harmonics, implemented in SCALE3 ABSPACK scaling algorithm.
Refinement. The refinement was carried out against all reflections. The conventional *R*-factor is always based on *F*. The goodness of fit as well as the weighted *R*-factor are based on *F* and *F*^2^ for refinement carried out on *F* and *F*^2^, respectively. The threshold expression is used only for calculating *R*-factors *etc*. and it is not relevant to the choice of reflections for refinement.The program used for refinement, Jana2006, uses the weighting scheme based on the experimental expectations, see _refine_ls_weighting_details, that does not force *S* to be one. Therefore the values of *S* are usually larger than the ones from the *SHELX* program.

### Fractional atomic coordinates and isotropic or equivalent isotropic displacement parameters (Å^2^)

**Table:**

	*x*	*y*	*z*	*U*_iso_*/*U*_eq_	
N1	−0.07910 (16)	−0.13039 (11)	0.82518 (8)	0.0261 (4)	
N2	0.05744 (16)	−0.14326 (11)	0.83819 (8)	0.0247 (4)	
N3	0.13057 (15)	−0.13457 (9)	0.76964 (8)	0.0195 (4)	
N4	0.37643 (15)	−0.06878 (9)	0.78252 (8)	0.0189 (4)	
N5	0.40183 (15)	0.08309 (9)	0.83954 (8)	0.0191 (4)	
N6	0.20270 (16)	0.15999 (10)	0.90914 (8)	0.0206 (4)	
N7	0.16374 (17)	0.16645 (11)	0.98552 (9)	0.0267 (5)	
N8	0.02387 (18)	0.16322 (11)	0.99042 (9)	0.0293 (5)	
C1	0.03639 (18)	−0.11614 (10)	0.71046 (10)	0.0190 (4)	
C2	−0.0979 (2)	−0.11366 (11)	0.74674 (10)	0.0211 (5)	
C3	−0.2245 (2)	−0.09657 (11)	0.70390 (10)	0.0234 (5)	
C4	−0.2101 (2)	−0.08297 (12)	0.62504 (10)	0.0253 (5)	
C5	−0.07273 (19)	−0.08592 (12)	0.58905 (11)	0.0251 (5)	
C6	0.0524 (2)	−0.10235 (12)	0.62995 (10)	0.0227 (5)	
C7	0.28645 (18)	−0.15303 (11)	0.76888 (9)	0.0199 (4)	
C8	0.35676 (18)	−0.01825 (11)	0.85796 (9)	0.0179 (4)	
C9	0.39816 (17)	0.00203 (11)	0.72380 (10)	0.0195 (4)	
C10	0.41143 (17)	0.09406 (11)	0.75863 (10)	0.0192 (4)	
C11	0.43808 (18)	0.17649 (12)	0.71390 (10)	0.0246 (5)	
C12	0.45606 (18)	0.16400 (13)	0.63336 (11)	0.0282 (5)	
C13	0.44790 (19)	0.07213 (14)	0.59973 (11)	0.0274 (5)	
C14	0.41670 (17)	−0.01080 (13)	0.64492 (10)	0.0228 (5)	
C15	0.44554 (18)	−0.06662 (11)	0.92129 (9)	0.0185 (4)	
C16	0.59542 (19)	−0.07058 (11)	0.91582 (10)	0.0211 (5)	
C17	0.6749 (2)	−0.11935 (13)	0.97213 (10)	0.0248 (5)	
C18	0.6050 (2)	−0.16512 (12)	1.03365 (10)	0.0259 (5)	
C19	0.4558 (2)	−0.16208 (13)	1.03928 (10)	0.0272 (5)	
C20	0.3761 (2)	−0.11246 (12)	0.98311 (10)	0.0238 (5)	
C21	0.35731 (18)	0.16107 (11)	0.89023 (10)	0.0217 (5)	
C22	0.08283 (18)	0.15079 (11)	0.86383 (10)	0.0196 (4)	
C23	−0.0315 (2)	0.15290 (11)	0.91632 (10)	0.0234 (5)	
C24	−0.1745 (2)	0.14330 (13)	0.89103 (11)	0.0275 (5)	
C25	−0.19652 (19)	0.13049 (12)	0.81287 (11)	0.0269 (5)	
C26	−0.0803 (2)	0.12856 (11)	0.75995 (10)	0.0241 (5)	
C27	0.06078 (18)	0.13921 (11)	0.78313 (10)	0.0207 (4)	
H3	−0.317263	−0.094484	0.728694	0.0281*	
H4	−0.294358	−0.071301	0.593908	0.0303*	
H5	−0.066743	−0.076054	0.533849	0.0301*	
H6	0.144966	−0.104274	0.605005	0.0273*	
H7a	0.309239	−0.203189	0.80607	0.0239*	
H7b	0.312986	−0.183038	0.720437	0.0239*	
H8	0.260422	−0.020574	0.878341	0.0215*	
H11	0.44408	0.240279	0.737235	0.0295*	
H12	0.474303	0.220137	0.601173	0.0339*	
H13	0.463773	0.065014	0.544828	0.0328*	
H14	0.408525	−0.074498	0.621619	0.0273*	
H16	0.643668	−0.039498	0.873004	0.0254*	
H17	0.778212	−0.121475	0.968596	0.0297*	
H18	0.660142	−0.199046	1.072481	0.0311*	
H19	0.407706	−0.19407	1.081737	0.0327*	
H20	0.272852	−0.109849	0.98703	0.0285*	
H21a	0.382104	0.222943	0.867254	0.026*	
H21b	0.412579	0.158613	0.937415	0.026*	
H24	−0.253694	0.145605	0.926964	0.0329*	
H25	−0.293096	0.122668	0.79372	0.0323*	
H26	−0.100448	0.119452	0.70565	0.0289*	
H27	0.139283	0.138788	0.746718	0.0248*	

### Atomic displacement parameters (Å^2^)

**Table:**

	*U*^11^	*U*^22^	*U*^33^	*U*^12^	*U*^13^	*U*^23^
N1	0.0243 (8)	0.0316 (7)	0.0223 (7)	−0.0027 (6)	0.0026 (6)	0.0007 (6)
N2	0.0248 (8)	0.0270 (7)	0.0224 (7)	−0.0032 (6)	0.0002 (6)	0.0027 (6)
N3	0.0199 (7)	0.0200 (6)	0.0186 (7)	−0.0020 (6)	0.0001 (5)	0.0009 (5)
N4	0.0206 (7)	0.0187 (6)	0.0173 (7)	−0.0004 (5)	−0.0010 (6)	0.0001 (5)
N5	0.0205 (7)	0.0163 (6)	0.0203 (7)	0.0005 (5)	−0.0014 (5)	−0.0007 (5)
N6	0.0235 (7)	0.0204 (6)	0.0178 (7)	0.0026 (5)	−0.0010 (6)	−0.0020 (5)
N7	0.0334 (9)	0.0276 (7)	0.0192 (7)	0.0056 (6)	0.0003 (6)	−0.0024 (6)
N8	0.0333 (9)	0.0311 (7)	0.0236 (8)	0.0049 (6)	0.0041 (6)	0.0020 (6)
C1	0.0212 (8)	0.0136 (6)	0.0224 (8)	−0.0009 (6)	−0.0026 (7)	−0.0005 (6)
C2	0.0228 (9)	0.0170 (7)	0.0234 (8)	−0.0018 (6)	0.0003 (7)	−0.0004 (6)
C3	0.0198 (8)	0.0211 (7)	0.0295 (9)	−0.0005 (6)	0.0009 (7)	−0.0011 (6)
C4	0.0252 (9)	0.0223 (8)	0.0283 (9)	0.0014 (7)	−0.0043 (7)	0.0013 (7)
C5	0.0262 (10)	0.0260 (8)	0.0231 (9)	0.0004 (7)	−0.0014 (7)	0.0018 (7)
C6	0.0237 (9)	0.0219 (7)	0.0227 (9)	0.0000 (6)	0.0014 (7)	0.0000 (6)
C7	0.0194 (8)	0.0164 (7)	0.0238 (8)	−0.0003 (6)	−0.0029 (6)	−0.0006 (6)
C8	0.0173 (8)	0.0181 (7)	0.0184 (8)	−0.0003 (6)	0.0009 (6)	−0.0001 (6)
C9	0.0138 (7)	0.0228 (7)	0.0219 (8)	0.0008 (6)	−0.0004 (6)	0.0034 (6)
C10	0.0139 (8)	0.0224 (7)	0.0214 (8)	0.0009 (6)	−0.0010 (6)	0.0018 (6)
C11	0.0205 (8)	0.0224 (7)	0.0308 (9)	−0.0013 (6)	−0.0019 (7)	0.0059 (7)
C12	0.0205 (9)	0.0334 (9)	0.0309 (10)	−0.0033 (7)	−0.0016 (7)	0.0129 (7)
C13	0.0192 (9)	0.0414 (9)	0.0215 (9)	0.0009 (7)	0.0000 (7)	0.0062 (7)
C14	0.0175 (8)	0.0306 (8)	0.0202 (8)	0.0016 (7)	−0.0008 (6)	0.0007 (7)
C15	0.0237 (8)	0.0162 (7)	0.0157 (8)	0.0006 (6)	−0.0007 (6)	−0.0013 (6)
C16	0.0240 (9)	0.0207 (7)	0.0188 (8)	−0.0019 (6)	0.0023 (7)	0.0000 (6)
C17	0.0234 (9)	0.0271 (8)	0.0238 (9)	0.0027 (6)	−0.0037 (7)	−0.0041 (7)
C18	0.0354 (10)	0.0241 (7)	0.0183 (8)	0.0046 (7)	−0.0045 (7)	0.0007 (6)
C19	0.0358 (10)	0.0257 (8)	0.0202 (8)	−0.0025 (7)	0.0033 (7)	0.0032 (7)
C20	0.0232 (9)	0.0250 (8)	0.0231 (9)	−0.0011 (7)	0.0033 (7)	−0.0005 (6)
C21	0.0211 (8)	0.0197 (7)	0.0241 (8)	0.0000 (6)	−0.0030 (7)	−0.0037 (6)
C22	0.0208 (8)	0.0149 (6)	0.0230 (8)	0.0020 (6)	−0.0009 (7)	0.0014 (6)
C23	0.0285 (9)	0.0180 (7)	0.0236 (8)	0.0034 (6)	0.0033 (7)	0.0028 (6)
C24	0.0233 (9)	0.0228 (8)	0.0362 (10)	0.0025 (7)	0.0078 (7)	0.0054 (7)
C25	0.0213 (9)	0.0203 (7)	0.0392 (11)	0.0000 (7)	−0.0026 (7)	0.0057 (7)
C26	0.0263 (9)	0.0187 (7)	0.0273 (9)	0.0013 (7)	−0.0039 (7)	0.0009 (6)
C27	0.0213 (8)	0.0195 (7)	0.0212 (8)	0.0018 (6)	0.0003 (7)	−0.0002 (6)

### Geometric parameters (Å, °)

**Table:**

N1—N2	1.298 (2)	C10—C11	1.385 (2)
N1—C2	1.379 (2)	C11—C12	1.405 (3)
N2—N3	1.365 (2)	C11—H11	0.96
N3—C1	1.364 (2)	C12—C13	1.382 (3)
N3—C7	1.467 (2)	C12—H12	0.96
N4—C7	1.440 (2)	C13—C14	1.403 (3)
N4—C8	1.480 (2)	C13—H13	0.96
N4—C9	1.412 (2)	C14—H14	0.96
N5—C8	1.479 (2)	C15—C16	1.394 (2)
N5—C10	1.402 (2)	C15—C20	1.391 (2)
N5—C21	1.436 (2)	C16—C17	1.387 (2)
N6—N7	1.364 (2)	C16—H16	0.96
N6—C21	1.470 (2)	C17—C18	1.389 (2)
N6—C22	1.363 (2)	C17—H17	0.96
N7—N8	1.300 (2)	C18—C19	1.387 (3)
N8—C23	1.380 (2)	C18—H18	0.96
C1—C2	1.393 (2)	C19—C20	1.392 (2)
C1—C6	1.404 (2)	C19—H19	0.96
C2—C3	1.405 (3)	C20—H20	0.96
C3—C4	1.375 (3)	C21—H21a	0.96
C3—H3	0.96	C21—H21b	0.96
C4—C5	1.417 (3)	C22—C23	1.392 (2)
C4—H4	0.96	C22—C27	1.411 (2)
C5—C6	1.375 (3)	C23—C24	1.402 (3)
C5—H5	0.96	C24—C25	1.370 (3)
C6—H6	0.96	C24—H24	0.96
C7—H7a	0.96	C25—C26	1.411 (3)
C7—H7b	0.96	C25—H25	0.96
C8—C15	1.516 (2)	C26—C27	1.375 (2)
C8—H8	0.96	C26—H26	0.96
C9—C10	1.397 (2)	C27—H27	0.96
C9—C14	1.378 (2)		
			
N2—N1—C2	108.32 (14)	C10—C11—H11	120.9899
N1—N2—N3	108.92 (13)	C12—C11—H11	120.9894
N2—N3—C1	109.99 (14)	C11—C12—C13	121.04 (17)
N2—N3—C7	118.83 (13)	C11—C12—H12	119.4794
C1—N3—C7	130.98 (14)	C13—C12—H12	119.4802
C7—N4—C8	116.31 (13)	C12—C13—C14	120.74 (17)
C7—N4—C9	120.85 (13)	C12—C13—H13	119.6288
C8—N4—C9	108.98 (12)	C14—C13—H13	119.6289
C8—N5—C10	109.28 (12)	C9—C14—C13	117.92 (16)
C8—N5—C21	118.80 (13)	C9—C14—H14	121.0407
C10—N5—C21	122.76 (13)	C13—C14—H14	121.0413
N7—N6—C21	118.06 (14)	C8—C15—C16	120.64 (14)
N7—N6—C22	109.87 (14)	C8—C15—C20	119.52 (15)
C21—N6—C22	132.05 (14)	C16—C15—C20	119.73 (15)
N6—N7—N8	108.92 (14)	C15—C16—C17	120.10 (15)
N7—N8—C23	108.32 (15)	C15—C16—H16	119.9515
N3—C1—C2	104.06 (14)	C17—C16—H16	119.9514
N3—C1—C6	133.79 (16)	C16—C17—C18	119.92 (17)
C2—C1—C6	122.15 (16)	C16—C17—H17	120.0389
N1—C2—C1	108.71 (15)	C18—C17—H17	120.0394
N1—C2—C3	130.20 (16)	C17—C18—C19	120.36 (16)
C1—C2—C3	121.09 (15)	C17—C18—H18	119.8197
C2—C3—C4	117.25 (16)	C19—C18—H18	119.819
C2—C3—H3	121.3774	C18—C19—C20	119.71 (16)
C4—C3—H3	121.3773	C18—C19—H19	120.1465
C3—C4—C5	120.93 (17)	C20—C19—H19	120.1462
C3—C4—H4	119.5339	C15—C20—C19	120.18 (17)
C5—C4—H4	119.5344	C15—C20—H20	119.9089
C4—C5—C6	122.70 (17)	C19—C20—H20	119.9072
C4—C5—H5	118.6524	N5—C21—N6	114.03 (13)
C6—C5—H5	118.6526	N5—C21—H21a	109.4706
C1—C6—C5	115.89 (16)	N5—C21—H21b	109.4714
C1—C6—H6	122.0553	N6—C21—H21a	109.4709
C5—C6—H6	122.0541	N6—C21—H21b	109.4714
N3—C7—N4	115.62 (12)	H21a—C21—H21b	104.4982
N3—C7—H7a	109.4709	N6—C22—C23	104.40 (14)
N3—C7—H7b	109.471	N6—C22—C27	133.65 (16)
N4—C7—H7a	109.4713	C23—C22—C27	121.94 (16)
N4—C7—H7b	109.4716	N8—C23—C22	108.49 (16)
H7a—C7—H7b	102.5346	N8—C23—C24	130.34 (17)
N4—C8—N5	102.31 (12)	C22—C23—C24	121.16 (16)
N4—C8—C15	111.06 (12)	C23—C24—C25	117.19 (17)
N4—C8—H8	114.7756	C23—C24—H24	121.4053
N5—C8—C15	114.04 (13)	C25—C24—H24	121.4053
N5—C8—H8	111.8387	C24—C25—C26	121.39 (17)
C15—C8—H8	103.2226	C24—C25—H25	119.3049
N4—C9—C10	108.75 (14)	C26—C25—H25	119.3045
N4—C9—C14	129.38 (15)	C25—C26—C27	122.55 (16)
C10—C9—C14	121.67 (15)	C25—C26—H26	118.7268
N5—C10—C9	108.88 (13)	C27—C26—H26	118.7256
N5—C10—C11	130.47 (15)	C22—C27—C26	115.76 (15)
C9—C10—C11	120.54 (15)	C22—C27—H27	122.1215
C10—C11—C12	118.02 (16)	C26—C27—H27	122.1225
			
?—?—?—?	?		

### Hydrogen-bond geometry (Å, °)

**Table:**

*Cg*6 is the centroid of the C15–C20 aromatic ring.

**Table:**

*D*—H···*A*	*D*—H	H···*A*	*D*···*A*	*D*—H···*A*
C12—H12···Cg6^i^	0.96	2.61	3.5597 (19)	169

Symmetry codes: (i) *x*+3/2, −*y*+1/2, −*z*+1.
